# Bis[2-(hy­droxy­imino)­cyclo­hexan-1-one oximato-κ^2^ *N*,*N*′]copper(II)

**DOI:** 10.1107/S160053681300785X

**Published:** 2013-04-05

**Authors:** Elena Melnic, Eduard B. Coropceanu, Lilia Croitor

**Affiliations:** a Institute of Applied Physics, Academy of Sciences of Moldova, Academiei str. 5, MD2028 Chisinau, Moldova; b Institute of Chemistry, Academy of Sciences of Moldova, Academiei str. 3, MD2028 Chisinau, Moldova

## Abstract

In the title compound, [Cu(C_6_H_9_N_2_O_2_)_2_], the Cu^II^ atom is located on an inversion center and has a square-planar environment. Two 2-(hy­droxy­imino)­cyclo­hexan-1-one oxim­ate monoanions chelate to the Cu^II^ atom and the Cu—N distances are 1.920 (3) and 1.930 (3) Å. There are two short intra­molecular O—H⋯O hydrogen bonds between the ligands. The complex mol­ecules stack into columns extended along the *c* axis, with a Cu⋯Cu distance between adjacent mol­ecules of 3.3060 (3) Å.

## Related literature

For complexes of copper(II) with 1,2-cyclo­hexa­ne­dione­di­oxime, see: Birkelbach *et al.* (1997 ▶); Cervera *et al.* (1997 ▶); Coropceanu *et al.* (2011 ▶); Mégnamisi-Bélombé & Endres (1983 ▶); Simonov *et al.* (1982 ▶). For the crystal structure of bis­(dimethyl­glyoximato-κ^2^ *N*,*N*′)nickel(II), see: Li *et al.* (2003 ▶).
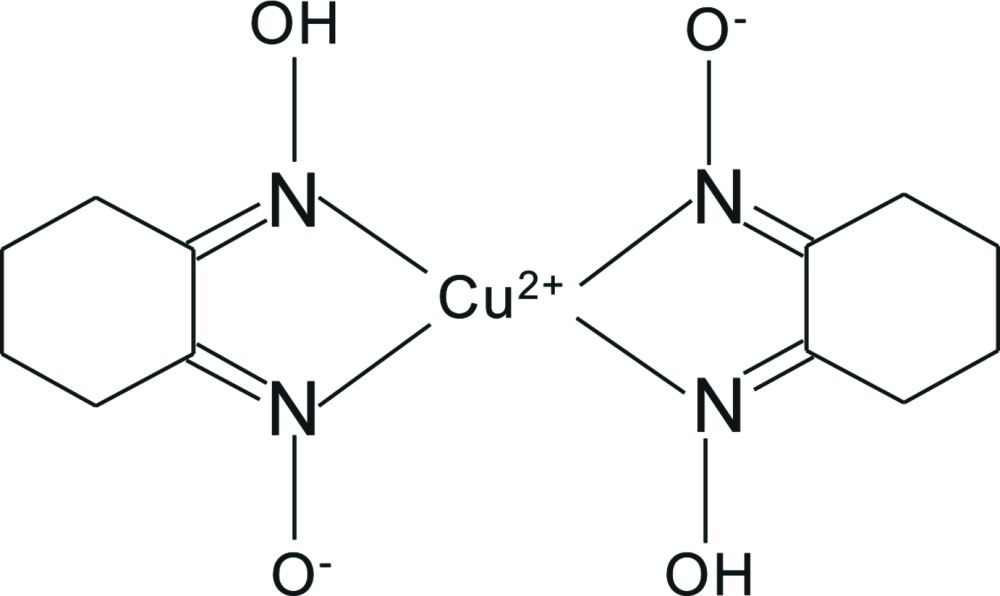


## Experimental

### 

#### Crystal data

[Cu(C_6_H_9_N_2_O_2_)_2_]
*M* *_r_* = 345.84
Monoclinic, 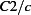

*a* = 20.8009 (12) Å
*b* = 10.1124 (7) Å
*c* = 6.6121 (5) Å
β = 100.787 (6)°
*V* = 1366.26 (16) Å^3^
*Z* = 4
Mo *K*α radiation
μ = 1.62 mm^−1^
*T* = 293 K
0.40 × 0.08 × 0.08 mm

#### Data collection

Oxford Diffraction Xcalibur Eos diffractometer
Absorption correction: multi-scan (*CrysAlis PRO*; Agilent, 2011 ▶) *T*_min_ = 0.878, *T*_max_ = 1.000
2458 measured reflections
1459 independent reflections
1013 reflections with *I* > 2σ(*I*)
*R*_int_ = 0.022

#### Refinement

*R*[*F* ^2^ > 2σ(*F* ^2^)] = 0.042
*wR*(*F* ^2^) = 0.109
*S* = 1.00
1459 reflections
101 parameters
H atoms treated by a mixture of independent and constrained refinement
Δρ_max_ = 0.31 e Å^−3^
Δρ_min_ = −0.30 e Å^−3^

### 

Data collection: *CrysAlis PRO* (Agilent, 2011 ▶); cell refinement: *CrysAlis PRO*; data reduction: *CrysAlis PRO*; program(s) used to solve structure: *SHELXS97* (Sheldrick, 2008 ▶); program(s) used to refine structure: *SHELXL97* (Sheldrick, 2008 ▶); molecular graphics: *SHELXTL* (Sheldrick, 2008 ▶); software used to prepare material for publication: *SHELXTL*.

## Supplementary Material

Crystal structure: contains datablock(s) I, global. DOI: 10.1107/S160053681300785X/gk2564sup1.cif

Structure factors: contains datablock(s) I. DOI: 10.1107/S160053681300785X/gk2564Isup2.hkl

Additional supplementary materials:  crystallographic information; 3D view; checkCIF report

## Figures and Tables

**Table 1:** Hydrogen-bond geometry (Å, °)

*D*—H⋯*A*	*D*—H	H⋯*A*	*D*⋯*A*	*D*—H⋯*A*
O1—H1*O*1⋯O2^i^	0.88 (6)	1.69 (6)	2.564 (4)	168 (6)

Symmetry code: (i) 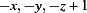.

**Fig. 1.**
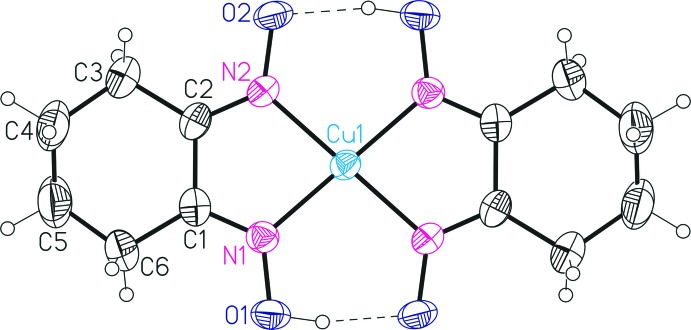
Molecular structure of the title compound. Displacement ellipsoids are shown at the 40% probability level. H atoms are presented as small spheres of arbitrary radius.

**Fig. 2.**
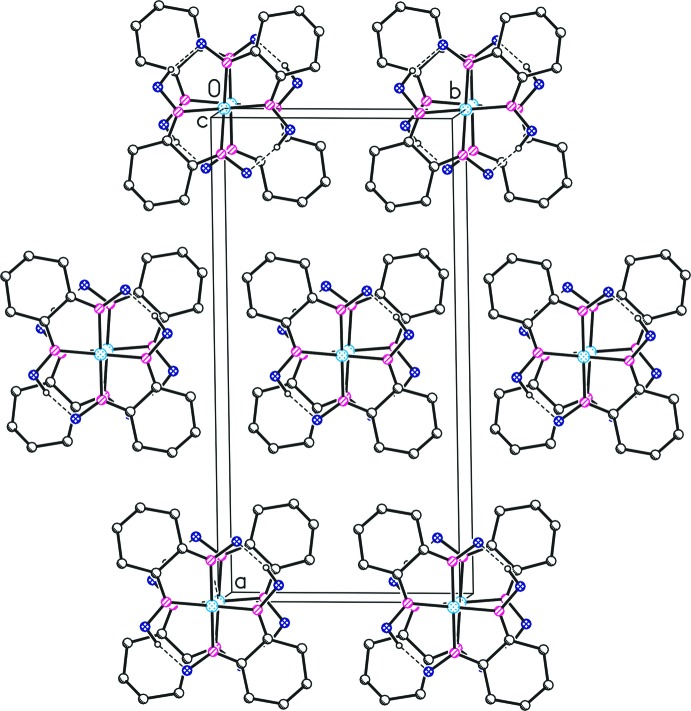
The crystal packing of the title compound.

## supplementary crystallographic information

### Comment

A large number of investigations on the complexation of copper(II) with 1,2-cyclohexanedionedioxime have been reported (Cervera *et al.*, 1997; Mégnamisi-Bélombé *et al.*, 1983; Simonov *et al.*, 1982; Birkelbach *et al.*, 1997; Coropceanu *et al.*, 2011). We report here the crystal structure of the title compound.

In the title coordination compound, the Cu^II^ atom has a square-planar geometry being coordinated by four N atoms from two monodeprotonated dioxime ligands (Fig.1). The monodeprotonated 1,2-cyclohexanedionedioxime coordinates in a typical bidentate mode through its oxime nitrogen atoms, thus leading to the formation of a five-membered chelate ring around the metal core with a N1—Cu1—N2 angle of 82.84 (12)° and slightly asymmetric Cu—N distances of 1.920 (3) and 1.930 (3) Å. The cyclohexyl ring of the 1,2-cyclohexanedionedioxime molecule adopts a half-boat conformation. Two dioxime residues are connected *via* O—H···O hydrogen bonds, typical for all bis-ligand complexes of α -dioximes (Table 1). The packing of the molecules involves columns of Cu atoms with a Cu—Cu separation of 3.3060 (3) Å (Fig.2). Despite the fact that the title compound crystallizes in a lower symmetry space group (C2/c) its crystal packing strongly resembles that of bis(dimethyl-glyoximato-κ^2^N,N')nickel(II) (Ibam) (Li *et al.*, 2003).

### Experimental

The title compound was obtained by disolving 0.02 g of copper acetate dihydrate and 0.028 g of 1,2-cyclohexanedionedioxime in the 30 ml methanol/dimethylformamide 1:5 (v/v) mixture. The resulting solution was boiled for *ca* 7 min, filtered off, and then slowly cooled to room temperature resulting in needle-shaped brown crystals (yield: 40%).

### Refinement

The C-bound hydrogen atoms were placed in calculated positions and were treated using a riding model approximation [*U*_iso_(H) = 1.2*U*_eq_(C)]. The O-bound hydrogen atoms were found from electron-density difference maps and refined freely.

### Crystal data

**Table:**

[Cu(C_6_H_9_N_2_O_2_)_2_]	*F*(000) = 716
*M**_r_* = 345.84	*D*_x_ = 1.681 Mg m^−^^3^
Monoclinic, *C*2/*c*	Mo *K*α radiation, λ = 0.71073 Å
Hall symbol: -C 2yc	Cell parameters from 636 reflections
*a* = 20.8009 (12) Å	θ = 3.1–28.8°
*b* = 10.1124 (7) Å	µ = 1.62 mm^−^^1^
*c* = 6.6121 (5) Å	*T* = 293 K
β = 100.787 (6)°	Needle, brown
*V* = 1366.26 (16) Å^3^	0.40 × 0.08 × 0.08 mm
*Z* = 4	

### Data collection

**Table:**

Oxford Diffraction Xcalibur Eos diffractometer	1459 independent reflections
Radiation source: Enhance (Mo) X-ray Source	1013 reflections with *I* > 2σ(*I*)
Graphite monochromator	*R*_int_ = 0.022
Detector resolution: 15.9914 pixels mm^-1^	θ_max_ = 27.0°, θ_min_ = 3.6°
ω scans	*h* = −26→20
Absorption correction: multi-scan (*CrysAlis PRO*; Agilent, 2011)	*k* = −12→12
*T*_min_ = 0.878, *T*_max_ = 1.000	*l* = −8→8
2458 measured reflections	

### Refinement

**Table:**

Refinement on *F*^2^	Primary atom site location: structure-invariant direct methods
Least-squares matrix: full	Secondary atom site location: difference Fourier map
*R*[*F*^2^ > 2σ(*F*^2^)] = 0.042	Hydrogen site location: inferred from neighbouring sites
*wR*(*F*^2^) = 0.109	H atoms treated by a mixture of independent and constrained refinement
*S* = 1.00	*w* = 1/[σ^2^(*F*_o_^2^) + (0.0427*P*)^2^ + 1.319*P*] where *P* = (*F*_o_^2^ + 2*F*_c_^2^)/3
1459 reflections	(Δ/σ)_max_ < 0.001
101 parameters	Δρ_max_ = 0.31 e Å^−^^3^
0 restraints	Δρ_min_ = −0.30 e Å^−^^3^

### Special details

**Table:**

Geometry. All esds (except the esd in the dihedral angle between two l.s. planes) are estimated using the full covariance matrix. The cell esds are taken into account individually in the estimation of esds in distances, angles and torsion angles; correlations between esds in cell parameters are only used when they are defined by crystal symmetry. An approximate (isotropic) treatment of cell esds is used for estimating esds involving l.s. planes.
Refinement. Refinement of F^2^ against ALL reflections. The weighted R-factor wR and goodness of fit S are based on F^2^, conventional R-factors R are based on F, with F set to zero for negative F^2^. The threshold expression of F^2^ > 2sigma(F^2^) is used only for calculating R-factors(gt) etc. and is not relevant to the choice of reflections for refinement. R-factors based on F^2^ are statistically about twice as large as those based on F, and R- factors based on ALL data will be even larger.

### Fractional atomic coordinates and isotropic or equivalent isotropic displacement parameters (Å^2^)

**Table:**

	*x*	*y*	*z*	*U*_iso_*/*U*_eq_	
Cu1	0.0000	0.0000	0.5000	0.0373 (2)	
O2	0.13049 (12)	−0.0982 (3)	0.6603 (4)	0.0539 (7)	
O1	−0.04254 (14)	0.2753 (3)	0.4482 (4)	0.0547 (7)	
N1	0.00765 (14)	0.1893 (3)	0.5076 (4)	0.0422 (7)	
N2	0.09204 (14)	0.0082 (3)	0.6185 (5)	0.0419 (7)	
C1	0.06607 (18)	0.2333 (4)	0.5736 (5)	0.0448 (9)	
C2	0.11585 (17)	0.1266 (4)	0.6355 (5)	0.0435 (9)	
C3	0.18591 (18)	0.1589 (4)	0.7027 (7)	0.0617 (11)	
H3A	0.2094	0.1347	0.5945	0.074*	
H3B	0.2035	0.1067	0.8235	0.074*	
C4	0.1975 (2)	0.3041 (5)	0.7532 (8)	0.0828 (15)	
H4A	0.1872	0.3214	0.8879	0.099*	
H4B	0.2435	0.3238	0.7602	0.099*	
C5	0.1582 (2)	0.3931 (5)	0.6018 (9)	0.0923 (17)	
H5A	0.1691	0.3772	0.4675	0.111*	
H5B	0.1695	0.4840	0.6396	0.111*	
C6	0.0852 (2)	0.3748 (4)	0.5874 (7)	0.0640 (12)	
H6A	0.0722	0.4137	0.7077	0.077*	
H6B	0.0621	0.4211	0.4669	0.077*	
H1O1	−0.077 (3)	0.224 (6)	0.408 (9)	0.15 (3)*	

### Atomic displacement parameters (Å^2^)

**Table:**

	*U*^11^	*U*^22^	*U*^33^	*U*^12^	*U*^13^	*U*^23^
Cu1	0.0342 (4)	0.0406 (4)	0.0358 (3)	0.0013 (3)	0.0032 (2)	−0.0015 (3)
O2	0.0417 (14)	0.0558 (16)	0.0611 (18)	0.0155 (14)	0.0019 (12)	0.0037 (14)
O1	0.0532 (17)	0.0465 (16)	0.0633 (19)	0.0142 (15)	0.0078 (13)	0.0032 (14)
N1	0.0428 (17)	0.0414 (17)	0.0418 (16)	0.0028 (15)	0.0062 (13)	−0.0024 (14)
N2	0.0343 (16)	0.0479 (18)	0.0419 (16)	0.0013 (15)	0.0029 (13)	0.0010 (14)
C1	0.050 (2)	0.048 (2)	0.038 (2)	−0.009 (2)	0.0122 (16)	−0.0028 (17)
C2	0.0383 (19)	0.059 (2)	0.0339 (19)	−0.0069 (19)	0.0071 (15)	−0.0046 (17)
C3	0.045 (2)	0.073 (3)	0.065 (3)	−0.010 (2)	0.0035 (19)	−0.003 (2)
C4	0.059 (3)	0.091 (4)	0.092 (4)	−0.029 (3)	−0.003 (2)	−0.003 (3)
C5	0.086 (4)	0.078 (3)	0.105 (5)	−0.037 (3)	−0.002 (3)	0.014 (3)
C6	0.067 (3)	0.049 (2)	0.075 (3)	−0.013 (2)	0.011 (2)	0.000 (2)

### Geometric parameters (Å, º)

**Table:**

Cu1—N1	1.920 (3)	C3—C4	1.515 (6)
Cu1—N1^i^	1.920 (3)	C3—H3A	0.9700
Cu1—N2	1.930 (3)	C3—H3B	0.9700
Cu1—N2^i^	1.930 (3)	C4—C5	1.475 (7)
O2—N2	1.338 (3)	C4—H4A	0.9700
O1—N1	1.359 (4)	C4—H4B	0.9700
O1—H1O1	0.88 (6)	C5—C6	1.514 (6)
N1—C1	1.291 (4)	C5—H5A	0.9700
N2—C2	1.293 (4)	C5—H5B	0.9700
C1—C6	1.483 (5)	C6—H6A	0.9700
C1—C2	1.499 (5)	C6—H6B	0.9700
C2—C3	1.478 (5)		
			
N1—Cu1—N1^i^	180.00 (17)	C2—C3—H3B	109.0
N1—Cu1—N2	82.85 (12)	C4—C3—H3B	109.0
N1^i^—Cu1—N2	97.15 (12)	H3A—C3—H3B	107.8
N1—Cu1—N2^i^	97.15 (12)	C5—C4—C3	113.4 (4)
N1^i^—Cu1—N2^i^	82.85 (12)	C5—C4—H4A	108.9
N2—Cu1—N2^i^	180.00 (7)	C3—C4—H4A	108.9
N1—O1—H1O1	104 (4)	C5—C4—H4B	108.9
C1—N1—O1	120.0 (3)	C3—C4—H4B	108.9
C1—N1—Cu1	114.9 (3)	H4A—C4—H4B	107.7
O1—N1—Cu1	125.1 (2)	C4—C5—C6	113.0 (4)
C2—N2—O2	121.5 (3)	C4—C5—H5A	109.0
C2—N2—Cu1	114.2 (2)	C6—C5—H5A	109.0
O2—N2—Cu1	123.9 (2)	C4—C5—H5B	109.0
N1—C1—C6	125.3 (4)	C6—C5—H5B	109.0
N1—C1—C2	113.7 (3)	H5A—C5—H5B	107.8
C6—C1—C2	120.9 (3)	C1—C6—C5	112.1 (4)
N2—C2—C3	124.8 (4)	C1—C6—H6A	109.2
N2—C2—C1	114.2 (3)	C5—C6—H6A	109.2
C3—C2—C1	121.0 (3)	C1—C6—H6B	109.2
C2—C3—C4	112.8 (4)	C5—C6—H6B	109.2
C2—C3—H3A	109.0	H6A—C6—H6B	107.9
C4—C3—H3A	109.0		

Symmetry code: (i) −*x*, −*y*, −*z*+1.

### Hydrogen-bond geometry (Å, º)

**Table:**

*D*—H···*A*	*D*—H	H···*A*	*D*···*A*	*D*—H···*A*
O1—H1*O*1···O2^i^	0.88 (6)	1.69 (6)	2.564 (4)	168 (6)

Symmetry code: (i) −*x*, −*y*, −*z*+1.
